# Microglia and Macrophages in the Pathological Central and Peripheral Nervous Systems

**DOI:** 10.3390/cells9092132

**Published:** 2020-09-21

**Authors:** Naoki Abe, Tasuku Nishihara, Toshihiro Yorozuya, Junya Tanaka

**Affiliations:** 1 Department of Anesthesia and Perioperative Medicine, Ehime University Graduate School of Medicine, Toon, Ehime 791-0295, Japan; abecometen422@yahoo.co.jp (N.A.); yorozuya@m.ehime-u.ac.jp (T.Y.); 2 Department of Molecular and cellular Physiology, Ehime University Graduate School of Medicine, Toon, Ehime 791-0295, Japan; jtanaka@m.ehime-u.ac.jp

**Keywords:** traumatic brain injury, brain infarction, carbon monoxide poisoning, peripheral nerve injury, NG2, macrophage

## Abstract

Microglia, the immunocompetent cells in the central nervous system (CNS), have long been studied as pathologically deteriorating players in various CNS diseases. However, microglia exert ameliorating neuroprotective effects, which prompted us to reconsider their roles in CNS and peripheral nervous system (PNS) pathophysiology. Moreover, recent findings showed that microglia play critical roles even in the healthy CNS. The microglial functions that normally contribute to the maintenance of homeostasis in the CNS are modified by other cells, such as astrocytes and infiltrated myeloid cells; thus, the microglial actions on neurons are extremely complex. For a deeper understanding of the pathophysiology of various diseases, including those of the PNS, it is important to understand microglial functioning. In this review, we discuss both the favorable and unfavorable roles of microglia in neuronal survival in various CNS and PNS disorders. We also discuss the roles of blood-borne macrophages in the pathogenesis of CNS and PNS injuries because they cooperatively modify the pathological processes of resident microglia. Finally, metabolic changes in glycolysis and oxidative phosphorylation, with special reference to the pro-/anti-inflammatory activation of microglia, are intensively addressed, because they are profoundly correlated with the generation of reactive oxygen species and changes in pro-/anti-inflammatory phenotypes.

## 1. Introduction

Microglia, resident macrophages in the central nervous system (CNS), are responsible for the clearance of degenerated cells and foreign materials from the CNS via phagocytosis, which establishes their status as immunocompetent cells. Although their origin has been a subject of debate for a long time, a fate-mapping analysis revealed that microglia originated from primitive macrophages present in the yolk sac, and not from myeloid cells [1]. In this sense, microglia are apparently different from macrophages or circulating monocytes. These differentiation processes of microglia are regulated by transcription factors such as the Runt-related transcription factor 1 (RUNX1), PU.1, and interferon regulatory factor 8 (IRF-8) [1,2].

In addition to phagocytosis, microglia display contradictory functions that have been described as a double-edged sword; i.e., pro- and anti-inflammatory effects, as well as neuroprotective and neurodestructive effects [3]. This heterogeneity of microglia is the result of modifications that occur via direct and indirect interactions with neurons and other glial cells, such as astrocytes and oligodendrocytes [4]. Moreover, macrophages that are recruited from the circulation and accumulate in and around the injury site play roles in CNS injuries that are accompanied by the disruption of the blood–brain barrier (BBB), thus rendering the pathology of CNS injuries even more complex. In recent years, to explain the heterogeneity of microglia, it has been broadly considered that the activation of microglia and macrophages is regulated by metabolic processes. In this review, we discuss the microglial metabolic processes in association with their heterogeneity in CNS injuries, to understand their complicated and conflicting functions. We also describe the analytic methods that are suitable for the identification of microglial heterogeneity and to differentiate them from blood-borne macrophages.

Microglia become activated in response to not only CNS injuries but also the peripheral nervous system (PNS) ones. The role of activated microglia in PNS injuries has been the subject of research in the field of pain [5]. The pathophysiology of PNS injuries is modified also by cells that have infiltrated the injured sites, such as macrophages and lymphocytes. Here, we discuss microglia in the spinal cord and macrophages in the injured peripheral nerve. These cells modulate the release of pro- and anti-inflammatory cytokines, as in CNS injuries, and engage in both neuroprotective and neurodestructive actions, thereby affecting nerve regeneration and pain behavior. Thus, microglia are critical players in various diseases and types of injury, both in the CNS and PNS, as they determine the course of the pathophysiology of the diseased/injured tissue. In the final section of this article, we describe therapeutic interventions targeting microglia/macrophages for pathological conditions of both the CNS and PNS.

## 2. Microglia in the Healthy Central Nervous System

Studies of microglia have long focused exclusively on their functions in the pathological brain and their ontogeny. Microglia in the normal brain, which are termed “resting microglia”, are supposedly on standby, waiting for the occurrence of a pathological event. However, during the last two decades, it has been found that microglia exhibit restless and vigorous movement in their delicate ramified processes even in the normal CNS [6]. Recently, these cells were renamed “homeostatic microglia”, because they play significant roles in the maintenance of homeostasis in the brain. In this section, we describe the physiological functions of microglia.

In the normal brain, microglia extend their processes toward synapses and are constantly surveilling synaptic activity. During this surveillance, microglia actively engulf synapses and control their number. These actions of microglia, which are termed “synaptic pruning”, are said to contribute to the formation of the neural circuits in the developing brain, as well as to homeostasis in the mature brain [4,6,7,8]. Previously, it was assumed that normal synaptic pruning is the result of competition between the activity levels and activity patterns of nearby neurons; however, it has been demonstrated that synaptic pruning by microglia depends on synaptic activity [9], which allows only active synapses to survive for the normal development of the neural circuits. Studies using cultured subventricular zone cells obtained from neonate mice revealed that neurogenesis is hampered by the depletion of microglia, but is reconstituted by adding microglia or the conditioned medium from microglia, suggesting that neurogenesis is modulated by soluble factors secreted from microglia [10]. In the pathological brain, for selective phagocytic elimination, microglia recognize degenerated materials that are opsonized with complements, the milk fat globule-EGF factor 8 (MFG-E8), and other factors, as discussed below. Microglia utilize a similar system for synaptic pruning in the developing brain [9,11,12,13].

ATP gradients and the P2Y12 receptor, which is a purinergic receptor, induce changes in this morphology and the expression of cathepsin S, a lysosomal enzyme [14]. Moreover, synaptic activity was enhanced in cathepsin S knockout mice, resulting in increased locomotor activity in individuals. These changes in microglia may be correlated with the phagocytic elimination of synapses. In fact, synaptic engulfment by microglia is more frequent at ZT0 compared with ZT12. Although it has long been known that synaptic strength and the number of synapses are reduced during sleep, the mechanisms underlying the synaptic loss observed during sleep remain obscure. These studies strongly suggest the involvement of microglia in the reduction of synapses. Glucocorticoids in circulation and noradrenaline in the brain appear to suppress the elimination of synapses, suggesting that circadian changes in these two substances are responsible for the morphological and functional circadian changes in microglia [13]. Interventions that increase glucocorticoids or noradrenaline in the brain cause insomnia while suppressing the activities of microglia, suggesting that microglia are involved in the induction of sleep. Sleep has been thought to be critical for brain development and plasticity [15,16], as well as for memory acquisition, reinforcement, and consolidation [17]. Considering the involvement of microglia in sleep, these cells are likely involved in memory. In fact, a recent report showed that microglial synaptic elimination by complement-dependent phagocytosis causes the forgetting of remote memories [18].

## 3. Microglia in the Pathological CNS

Primary injuries in the CNS, such as vascular accidents and mechanical brain injuries, cause damages in neuronal cells and their processes. The primary injuries then cause secondary ones that spread beyond the primary lesions via inflammation and other mechanisms [19,20]. Secondary injuries include (1) neuronal damage caused by the excitotoxicity of glutamate released from neurons and microglia; (2) chemical modification of proteins, phospholipids, and nucleic acids in neurons by reactive oxygen species (ROS); (3) neuronal damage caused by excessive inflammation associated with local and systemic immune reactions; (4) neuronal cell death caused by excessive phagocytosis by immune cells. These secondary injuries are deeply related to microglia [21]. In this section, we describe the roles of microglia in various CNS injuries.

### 3.1. Heterogeneity of Microglia in Traumatic Brain Injury and Cerebral Infarction

**Figure 1:**
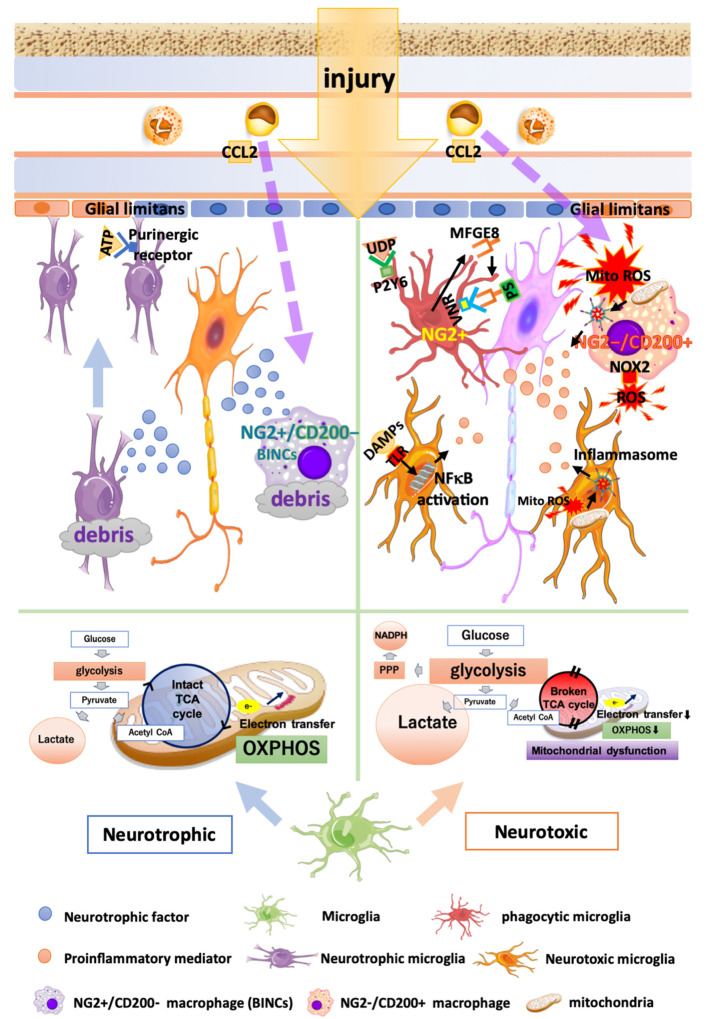
Multiple functions of microglia and macrophages in brain injury. Neurotrophic microglia and macrophages (left half) exhibit an intact TCA cycle and stable mitochondrial OXPHOS. Microglia that have migrated to the injury site release anti-inflammatory cytokines and neurotrophic factors, thus encouraging wound healing and debris clearance. Neuroprotective infiltrated macrophages called BINCs (brain Iba1+/NG2+ cells) express a variety of neuroprotective factors. Neurotoxic microglia and macrophages (right half) produce energy in a glycolysis-dependent manner and exhibit increased lactate production, glucose uptake, and pentose phosphate pathway (PPP). DAMPs recognized by TLR stimulate NFκB pathways, leading to an increased expression of proinflammatory mediators. Microglia phagocytose viable neurons by recognizing opsonized PS via VNRs and the humoral “eat-me” signal UDP, through P2Y6. Phagocytic microglia and macrophages express the phagocytosis marker CD68 and NG2. Neurotoxic infiltrated macrophages (NG2^−^/CD200^+^ macrophages) release a greater amount of MitoROS, IL1β, and NOX2 compared with microglia. MitoROS not only directly damages the brain tissues, but also induces proinflammatory reactions by inducing the formation of inflammasomes.

Traumatic brain injury (TBI) and cerebral infarction cause the rapid activation of microglia [22]. The responses of microglia in TBI and cerebral infarction are characterized by morphological differentiation [23,24,25], migration [26], and phagocytosis [27], as well as the release of bioactive substances, such as cytokines/chemokines [28], ROS [29,30], and neuroprotective factors [25]. Whether microglia in severely injured brains are neurotoxic or neuroprotective is a matter of debate. The multiple functions of microglia in brain injuries are shown in Figure 1.

Damaged cells release damage-associated molecular patterns (DAMPs). Microglia become activated after recognizing DAMPs via Toll-like receptors (TLRs) and nucleotide-binding oligomerization domain-like receptors (NLRs) [31,32]. After activation, microglia generate proinflammatory mediators, such as interleukin 1β (IL1β), IL6, IL12, tumor necrosis factor α (TNFα), and nitric oxide (NO). They also release neuroprotective factors, such as the insulin-like growth factor 1 (IGF1) and anti-inflammatory cytokine transforming growth factor β1 (TGFβ1) [25]. The polarization of microglia, i.e., the M1 and M2 activated microglia, has also been investigated. This classification is based on distinctive gene expression patterns of macrophages cultured with either interferon γ (IFNγ)/lipopolysaccharide (LPS) or IL4 [33]. It had long been believed that M1 (or classically activated) microglia engaged in the release of proinflammatory cytokines and ROS, whereas M2 (or alternatively activated) microglia released anti-inflammatory cytokines, thereby stimulating wound healing and debris clearance [25,34]. Therefore, various studies have been conducted based on this classification. However, Butovsky et al. [35] indicated the differences in gene expression signatures among adult microglia, neonate microglia, monocytes, and BV2 cell lines using gene and microRNA array analysis and quantitative proteomic analysis. In addition, as shown in the studies reported by Marciano et al. [36] and Lambertsen et al. [37], because microglia and myeloid cells are not exposed to only one specific cytokine in vivo in the pathological brain, such as the TBI and cerebral infarction conditions, heterogeneous microglial populations exist that have varying degrees of M1- and M2-type characteristics [38,39]. The existence of microglial heterogeneity has also been shown in an experiment using single-cell RNA sequencing [40]. Thus, it is impossible to explain the diversity of microglia in TBI and cerebral infarction based on this simple classification [38].

### 3.2. Metabolic Changes in Microglia in TBI and Infarction

In recent years, it has been broadly considered that metabolic processes regulate the activation and heterogeneity of microglia and macrophages [41]. Glucose is metabolized into pyruvic acid via glycolysis in the cytoplasm and is then efficiently utilized by the tricarboxylic acid (TCA) cycle in mitochondria. The NADH and FADH_2_ obtained from the TCA cycle act as proton donors for mitochondrial electron transport and aerobic/mitochondrial respiration (oxidative phosphorylation; OXPHOS). In this collective process, 36 ATP molecules are generated per glucose molecule. Under anaerobic conditions, energy production by OXPHOS becomes impossible, and energy is produced by anaerobic glycolysis while synthesizing lactate. Activated pro-inflammatory microglia and macrophages produce ATP in a glycolysis-dependent manner even under aerobic conditions, and increase lactate production and glucose uptake [42,43,44,45]. This enhanced glycolytic pathway and increased glucose uptake under aerobic conditions are known as the Warburg effect [46]. An intact TCA cycle and stable mitochondrial OXPHOS are required for the acquisition of anti-inflammatory and tissue-reparative phenotypes by microglia and macrophages to [43,45]. The administration of the C-X3-C motif chemokine ligand 1 (CX3CL1), also known as fractalkine, to murine models of cerebral infarction, suppresses the expression of genes related to the glycolytic pathway, upregulates genes related to OXPHOS, and changes microglia toward an anti-inflammatory population [47]. Thus, the use of glycolysis or OXPHOS as the main energy source may be a critical determinant of the pro- or anti-inflammatory phenotypes of microglia. The pentose phosphate pathway (PPP), which is a collateral metabolic pathway of glycolysis, generates NADPH. In turn, NADPH supplies electrons to NADPH oxidase (NOX), to generate ROS [42,45]. The blockage of glucose-6-phosphate dehydrogenase (G6PDH), which is the rate-limiting enzyme of the PPP, suppressed ROS production in LPS-stimulated mesencephalic neuron–glia cultures [48]. Thus, the functional heterogeneity of microglia is profoundly correlated with changes in energy metabolism.

### 3.3. Phagocytosis by Microglia and Find-Me/Eat-Me Signals in TBI and Infarction

When TBI or cerebral infarction occurs, degenerating cells release extracellular nucleotides called find-me signals, such as ATPs [49] and humoral factors including chemokines and sphingosine-1-phosphate [50]. These substances transmit signals to their neighboring microglia, for enhanced migration to the injury sites. This migration is mediated by purinergic receptors (P2Y6, P2Y12, and P2X4), the tyrosine-protein kinase receptor (Tyro3), and the Axl receptor tyrosine kinase (Axl). Purinergic receptors interact with extracellular nucleotides. Tyro3 and Axl interact with growth-arrest specific protein 6 (GAS6), which is known as an eat-me signal molecule [26,51]. Microglia extend their processes to the injury site, thus preventing the spread of injury by reinforcing the glial limitans that are collapsed by trauma or ischemia [26,52]. The inhibition of the find-me signal-associated microglial migration exacerbates brain injury in TBI models; therefore, microglial migration may be necessary to prevent the spread of the injury [26,52]. Microglia at lesions sense eat-me signal molecules, such as phosphatidylserine (PS), MFG-E8, complements, and GAS6, via phagocytic receptors, including the vitronectin receptor (VNR), brain-specific angiogenesis inhibitor 1 (BAI1), MER receptor tyrosine kinase (MerTK), and complement receptors; subsequently, the cells internalize degenerating cells in and around lesions via eat-me signals [27]. The clearance of degenerating cells and debris by phagocytosis during the acute phase in the trauma and infarction may be beneficial for the repair and regeneration of the injured tissues; however, excessive and long-lasting phagocytosis is likely to aggravate the injuries by eliminating neurons that are still viable. The phagocytic elimination of viable cells by phagocytic cells is called phagoptosis [53]. Microglial phagoptosis is caused by reversible exposure of PS to the extracellular space in viable neurons. This is induced by the activation of PS scramblase, which is probably the same as the transmembrane protein 16 (TMEM16), and the suppression of PS translocase (probably type 4 P-type ATPases) by ROS and the decrease in intracellular ATP levels in neurons. The exposed PS is opsonized by the MFG-E8 secreted from activated microglia. Microglia are thought to recognize viable neurons exposing PS via VNRs and MerTK, resulting in phagocytic elimination of the neurons [53,54,55,56,57]. Microglia that cause phagoptosis express a possible phagocytosis marker, the neural/glial antigen 2 (NG2) chondroitin sulfate proteoglycan, and possess large phagosomes that express CD68 [58]. These microglia are observed exclusively at the regions neighboring the lesion cores. Why these cells express NG2 or the specific roles of NG2 in the phagocytic microglia remain to be clarified.

The humoral eat-me signal uridine 5′-diphosphate (UDP) and its receptor, the P2Y6 receptor, may be involved in phagoptosis [59,60,61]. Injured neurons leak not only ATP, a find-me signal, but also uridine 5′-triphosphate (UTP). As the concentration of UTP is lower than that of ATP, it does not spread to a wider area compared with ATP. The leaked UTP is rapidly metabolized to UDP and becomes an eat-me signal. Microglia sense ATP via the P2Y12 receptor and migrate to the vicinity of the damaged cells [49,62], where they then sense UDP via the P2Y6 receptor and initiate phagocytosis. However, unlike other eat-me signals, such as PS, UDP is a humoral factor; therefore, excessive secretions from the injured nerve diffuse widely beyond the degenerated tissues. Hence, recognition by microglia may become unreliable, resulting in the induction of phagoptosis. However, naturally, neurons and other living cells have don’t-eat-me signals, such as CD47, on their surfaces and are protected from phagocytosis. CD47 interacts with the signal-regulatory protein α (SIRPα) expressed in microglia and macrophages, while mediating suppressive signals for phagocytosis secreted by microglia and macrophages [63,64]. Therefore, phagoptosis may occur based on the synergistic actions of suppressed don’t-eat-me signals and enhanced eat-me signals. Further investigations are required to clarify the mechanisms underlying the permission and suppression of phagoptosis.

### 3.4. Oxidative Stress Caused by Microglia and Macrophages in TBI and Infarction

Oxidative stress caused by ROS in the acute phase of TBI and cerebral infarction is thought to be detrimental, and macrophages and microglia have been recognized as the main cells that produce ROS [29]. Cells contain various sources of ROS. NOX are well known as enzymatic systems that actively generate ROS [29]. The neurotoxicity of NOX-derived ROS is evident because knockout or inhibition of the *NOX* gene or its activities reduced the damaged area and improved neurological prognoses in models of TBI and cerebral infarction [65,66,67]. However, more than 90% of the ROS produced by cells originate from mitochondria (MitoROS) [68], which are thought to be the main cause of the oxidative damage that occurs during brain injury [69,70]. Mitochondria consume more than 90% of the total respired oxygen and generate ROS from 2% of that oxygen [71]. The inflammatory stimuli mediated by TLR receptors and other factors cause impairment of the mitochondrial electron transport chain, leading to ROS generation accompanied by reduced mitochondrial ATP production [72,73,74]. The MitoROS generated during the acute phase of the brain injury not only directly damage the brain tissues, but also induce pro-inflammatory reactions [75] through the formation of inflammasomes. MitoROS activate the nucleotide-binding oligomerization domain-like receptor family, pyrin domain-containing 3 (NLRP3), and the activated NLRP3 forms a 7-mer together with adaptor proteins, such as apoptosis-associated speck-like protein containing a CARD (ASCs) and pro-caspase-1, thus constructing inflammasomes of the bulky protein complexes. The inflammasomes activate caspase-1, which cleaves pro-IL1β into active IL1β, to play a central role in inflammation in TBI and infarction [76]. In the acute phase of brain injury, blood-borne macrophages release much greater quantities of MitoROS than do microglia and express mRNAs encoding IL1β and NOX2; NOX2 is another important source of ROS [70]. Conversely, activated and resting microglia expressed higher levels of the mRNAs of potentially neuroprotective factors, such as TGFβ1 [70]. TGFβ1 inhibits the translocation of NFκB into cell nuclei by persistently inhibiting the phosphorylation of IκB kinase induced by TLR ligands [77]. Moreover, TGFβ1 inhibits the phosphorylation of the signal transducer and activator of transcription 1 (STAT1) and the expression of IRF1, causing the suppression of the proinflammatory response of microglia. These effects of TGFβ1 may result in neuroprotection in injured brain tissues [77,78]. Interventions that increase the actions of TGFβ1 in stroke models have been shown to ameliorate the outcomes of these animals [78]. Taken together, these findings suggest that both activated and resting microglia may exert neuroprotective effects in the acute phase of TBI and cerebral infarction. The administration of the colony-stimulating factor 1 (CSF1, or macrophage colony-stimulating factor; M-CSF) receptor antagonist PLX3397 to a murine stroke model depletes microglia. In turn, the elimination of microglia by PLX3397 increases infarct volume, indicating that the overall effects of microglia on ischemic injury are ameliorative [79]. These results suggest that the neuroprotective actions of microglia may be overwhelming against their potentially harmful effects. Recently, Krasemann et al. [80] and Keren-Shaul et al. [81] identified gene signatures of a microglial subpopulation specific to neurodegenerative disease using single-cell sequencing of microglia. Hence, in the treatment of TBI and infarction, single-cell sequencing, considering the spatial and temporal varieties of microglia in the lesions may represent a breakthrough in further evaluating the function of microglia at the molecular level.

### 3.5. Heterogeneity of Blood-Borne Macrophages in TBI and Infarction

Together with microglia, blood-borne macrophages play a critical role in brain pathologies that exhibit BBB disruption. The multiple functions of macrophages in brain injuries are shown in Figure 1. It has long been difficult to distinguish microglia from macrophages because of the similarities in their morphology, function, and antigen expression [58]. However, it is now becoming easier to separate and analyze them because of the development of flow cytometry and the identification of microglia-specific genes, such as *CX3CR1*, G protein-coupled receptor 34 (*Gpr34*), P2Y12 receptor, P2Y13 receptor, Siglec H, Tmem119, and triggering receptor expressed on myeloid cells 2 (*Trem2*) [35,82,83]. In the pathological brain with BBB disruption, such as TBI and cerebral infarction, neuronal and microglial cell death occurs rapidly in the lesion core, accompanied by the infiltration of leukocytes, such as neutrophils, monocytes, and lymphocytes [84]. Most macrophages in brain lesions with BBB disruption express NG2 on their plasma membrane. These cells were initially thought to be microglia; however, it has been demonstrated that they are blood-borne macrophages based on experiments of transplantation of the bone marrow from rats with ubiquitous expression of the enhanced green fluorescent protein [85]. NG2^+^/CD200^−^ macrophages and NG2^−^/CD200^+^ macrophages accumulate at the lesion core of the pathological brain with BBB disruption [86]. The CD200 expressed by neurons binds to macrophages expressing the CD200 receptor while inhibiting the inflammatory response of macrophages [87,88,89]; however, macrophages expressing CD200 (NG2^−^/CD200^+^ macrophages) release proinflammatory mediators, such as ROS and IL1β, thus acting as a detrimental player [86]. In a rat TBI model, 8-hydroxy-2′-deoxyguanosine (8-OHDG) accumulated in the nuclei of these detrimental macrophages, suggesting the damage to DNA and the resultant degeneration of the macrophages, probably by ROS produced by themselves, during the acute phase [70]. The surviving macrophages express NG2 (NG2^+^/CD200^−^ macrophages) in the subacute phase and proliferate rapidly. These macrophages are called brain Iba1^+^/NG2^+^ cells (BINCs) [84]. The transplantation of BINCs isolated from the infarcted brains of the rats into the ischemic lesions of other rats led to an abundant proliferation of BINCs and the amelioration of the prognosis of the transplanted rats [85]. BINCs have been shown to exhibit high expression levels of neuroprotective factors, such as IGF-1 and the hepatocyte growth factor (HGF), and to prevent the exacerbation of the tissue damage caused by injury and ischemia [85,90].

### 3.6. Carbon Monoxide Poisoning

Carbon monoxide (CO) inhalation causes prolonged serious dysfunctions of the CNS. After recovery from the impaired consciousness that occurs in the acute phase, it causes delayed encephalopathy with the decline in cognitive function being the main symptom that is observed weeks to months later. The main cause of this intoxication has been thought to be demyelination; therefore, this injury can also be considered a demyelinating disease. The major cause of this serious condition may be tissue hypoxia caused by the strong binding of CO to hemoglobin. However, many of its events are not explained by hypoxia alone; thus, many unclear issues remain, such as the pathogenic mechanism underlying the delayed encephalopathy, the causes of demyelination, and the differences from pure hypoxia.

In the lesions of multiple sclerosis, which is a typical demyelinating disease, microglia and macrophages are activated and engage in myelin removal and regeneration, while inflammation spreads over surrounding tissues [91]. Microglial activation was similarly observed after CO poisoning, suggesting the involvement of these cells in the delayed encephalopathy caused by this insult [92,93]. However, our recent study proposed a unique hypothesis regarding the pathogenesis of the CO-poisoning-induced encephalopathy. Compared with the pure hypoxia-induced brain injury that accompanies marked microglial activation, the CO-poisoning model is characterized by a reduced number of microglia, as revealed by immunohistochemistry, RT-PCR, Western blotting, and flow cytometry [94]. These data are consistent with previous studies showing that, among glial cells, microglia and oligodendrocytes are the most vulnerable to ischemia [95]. In particular, microglia in cerebral infarction models undergo degeneration within hours after the onset of an ischemic event [84].

Microglia may play neuroprotective roles by releasing neurotrophic factors, such as IGF1, IGF2, HGF, the fibroblast growth factor 2 (FGF2), the brain-derived neurotrophic factor (BDNF), and the platelet-derived growth factor AA (PDGF-AA). Through the secretion of these neuroprotective factors, microglia promote the survival and maturation of oligodendrocyte precursor cells (OPCs)/NG2 glia and neurons [96,97,98,99,100]. In the hippocampal tissues of the CO-poisoning model, the mRNAs of these neurotrophic factors are downregulated for three weeks after CO inhalation; moreover, this mRNA downregulation is attributable to the decrease in mRNA expression by the microglia isolated from the hippocampus [94]. The reduced expression of neuroprotective factors by microglia may be responsible for the suppressed restoration of other glial cells, especially oligodendrocytes, in addition to the impairment of neuronal activities. Conversely, pure hypoxia results in the upregulation of the mRNAs of some neuroprotective factors, such as IGF1 and HGF, in the hippocampal tissues. These findings, together with the highly reduced number of microglia observed in the CO-poisoning model and the maintained survival of microglia in the pure hypoxic model, indicate the significant beneficial effects of microglia on the injured brains.

Interestingly, it has been suggested that CO poisoning not only damages mature neurons and glial cells but also reduces their progenitors. In particular, the reduction of OPCs/NG2 glia may be correlated with the prevention of the restoration of myelin, as well as demyelination. Interventions that support the survival of microglia under CO intoxication, to enhance the survival of oligodendrocytes and their progenitors and the restoration of myelin, are promising therapies against CO poisoning.

## 4. Microglia/Macrophages in the PNS Injury

In the neuroinflammation that follows peripheral nerve injury, macrophages and microglia play a central role at the injury site and in the spinal cord, respectively. Here, we describe the functions of macrophages and microglia in peripheral nerve injury.

Macrophages accumulate at sites of peripheral nerve injury by the stimulation of the C-C motif chemokine ligand 2 (CCL2), also known as monocyte chemoattractant protein 1 (MCP1). Macrophages stimulate nerve regeneration by phagocytosing degenerated tissues and cells [101]. As mentioned above, macrophages express NG2 on their plasma membrane in the infarct and injury sites of the CNS, being not only phagocytic but also neuroprotective by expressing various neurotrophic factors [85,90]. In addition, NG2 expression on macrophages is also observed in PNS injuries [102]. It is reported that the extracellular domain of NG2 on macrophages is shed by their expressing matrix metalloproteinase 14 (MMP14) in peripheral nerve injury [102]. Local injection of an MMP14 inhibitor into the site of peripheral nerve injury results in the retention of the NG2 protein in peripheral nerve tissues and the concomitant increase in the rate of nerve regeneration, suggesting that the NG2 protein and MMP14 play a critical role in the enhancement of nerve regeneration [102].

**Figure 2:**
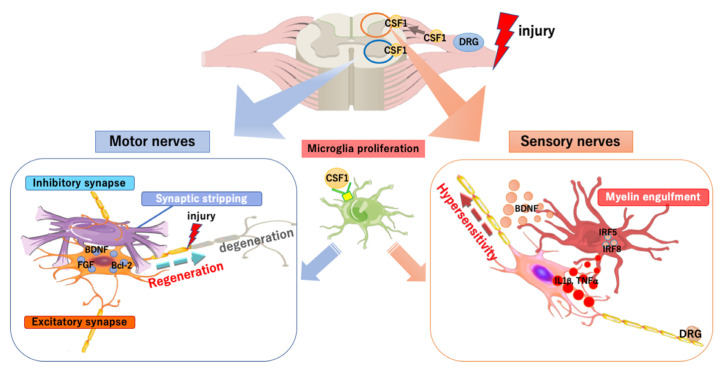
Functions of spinal microglia in peripheral nerve injury. When the peripheral nerve is injured, the colony-stimulating factor 1 (CSF1) is rapidly induced in DRG neurons. CSF1 transported to the spinal dorsal horn acts on the CSF1 receptor (CSF1R) of microglia, for their proliferation and activation. In the dorsal horn, the interferon regulatory factor 5 (IRF5) and IRF8, which are transcription factors, are induced in the activated microglia, followed by the release of several cytokines (including the tumor necrosis factors α, interleukin 1β, and the brain-derived neurotrophic factor), via which hypersensitivity is induced and maintained. Microglia activated by CSF1 in the ventral horn block signals from inhibitory synapses by synaptic stripping and induce the expression of several factors, which stimulates the degeneration and regeneration of injured nerves.

Peripheral nerve injury causes microglial activation in the spinal cord far from the injury site (schema in Figure 2). To date, microglial activation in the spinal dorsal horn has been intensively studied to show the important role of these cells in the development and maintenance of neuropathic pain [5]. Microglial activation in the dorsal horn upon peripheral nerve injury is initiated by CSF1. When the peripheral nerve is injured, CSF1 expression is rapidly induced in dorsal root ganglion (DRG) neurons and the protein is transported to the dorsal horn. Microglia expressing the CSF1 receptor proliferate to form microgliosis upon receiving the CSF1 protein [103]. Simultaneously, the DNAX activating protein of 12 kDa (DAP12), which is a membrane adaptor protein, is upregulated and is involved in the activation of microglial cells and the development of allodynia. The signaling increases the expression of transcription factors such as IRF8 and IRF5, and subsequently induces and maintains neuropathic pain by triggering the expression of receptors, such as P2RX4 and CX3CR1, and cytokines, such as TNFα, IL1β, and BDNF [104].

Microglial activation is found not only in the dorsal horn but also in the ventral horn. Microglial proliferation in the ventral horn is similarly induced by CSF1 [105]. However, their activation mode seems to be different in the ventral and dorsal horns of the spinal cord. In the dorsal horn, microglia exhibit an amoeboid morphology, a classic mode of activation, whereas in the ventral horn, they display an elongated morphology surrounding neurons that appear to engage in synaptic stripping [44]. Synaptic stripping was first reported in a facial nerve injury model in 1968 by Blinzinger et al. [106]. Although such microglia have been thought to remove synaptic input by synaptic stripping [107], a recent study using an axotomy model showed that reduced synaptic input precedes synaptic stripping in the injured motor nerve; microglia prevent synaptic input chemically by releasing ATP and adenosine, followed by a physical blockade by synaptic stripping [108]. During this process, microglia are thought to exert neuroprotective effects by releasing a variety of neurotrophic factors [109], blocking signals from inhibitory synapses, and inducing the expression of antiapoptotic and neuroprotective factors, such as Bcl-2, FGF2, and BDNF, in neurons [110]. Thus, microglia enhance the regeneration of axon terminals in injured nerves and synaptogenesis [108]. Synapse removal and remodeling occur even in the absence of microglial proliferation [105]. However, the activation of spinal microglia in the ventral and dorsal horns after peripheral nerve injury accompanies the upregulation of proinflammatory cytokines, IL1β, and IL6. Arg1 is increased in the dorsal horn, whereas there is no change in the expression of CD206 and YM1. Therefore, peripheral nerve injury-induced activated microglia cannot be clearly divided into classifications [44].

After peripheral nerve injury, microglia in the ventral horn engulf synapses and those in the dorsal horn engulf myelin components [44]. The expression of mRNAs for the phagocytic markers CD68 and F4/80 in the ventral horn was lower than that in the dorsal horn, suggesting that microglia in the dorsal horn possess stronger phagocytic activity than do those in the ventral horn [44]. The phagocytosis of myelin by microglia in the dorsal horn may be correlated with the development of neuropathic pain.

Much evidence has been accumulating regarding the involvement of spinal microglia in the pathophysiological process in peripheral nerve injury. However, many questions remain unanswered; i.e., why are there differences in the reactivity of microglia of the ventral and dorsal horns? Why is microglial encirclement observed only in the ventral horn motor neurons? Activated microglia in the ventral horn appear to be more neuroprotective than those in the dorsal horn because motor dysfunction starts to ameliorate within three days after the onset of peripheral nerve injury, whereas neuropathic pain persists for much longer periods [44]. If the phenotypic changes that occur in the ventral microglia can be induced in the dorsal microglia, the persistent neuropathic pain may be ameliorated.

## 5. Therapeutic Approaches Targeting Microglia

As mentioned above, excessive inflammation caused by activated microglia and macrophages can worsen the pathological courses in the injured CNS. Therapeutic interventions that can control the inflammation and/or transform microglia and macrophages into neuroprotective phenotypes have long been sought; however, there are currently no clinically applicable interventions that control microglia and macrophage functions. This might be at least partly attributed to the diversity of microglia and macrophages. Among the various reported agents that control microglia/macrophage functions in laboratory settings, we discuss here some therapeutic strategies regarding the modulation of immunoreactions and the metabolism of microglia.

### 5.1. Immunomodulation

#### 5.1.1. Suppressing the Proinflammatory Reaction of Microglia/Macrophages

Glucocorticoids have strong immunosuppressive effects on many types of cells and are the most commonly used anti-inflammatory agents in clinical settings. The anti-inflammatory effects of glucocorticoids on microglia are more potent compared with those of ibuprofen, indomethacin, minocycline, and statins. Dexamethasone (Dex), a synthetic glucocorticoid that is a specific ligand for the glucocorticoid receptor, strongly inhibits the LPS-induced release of microglial NO and the expression of the IL1β and TNFα mRNAs [111]. When primary neurons are co-cultured with primary microglia in the presence of LPS, the NO released from microglia causes the degeneration of neurons. However, the administration of Dex can prevent this neurodegeneration almost completely. Moreover, Dex increases the expression of the mRNA of the neuroprotective factors HGF and IGF-1 [112]. The knockdown of the expression of the glucocorticoid receptor in the cerebral infarction model mice led to an increase in the expression of proinflammatory cytokines and infarction size [113]. Moreover, the administration of glucocorticoids restored BBB integrity and alleviated cerebral edema in TBI model mice [114]. However, despite these results, clinical trials employing treatments with glucocorticoids for cerebral infarction and TBI have not been successful [115,116]. This may be caused by the degradation of glucocorticoid receptors by proteasomes in the lesions [117]. Glucocorticoids have various adverse effects, such as increased incidence of the infection and impaired glucose tolerance. These unfavorable effects may hinder its application to control microglia and macrophages in the CNS.

Glucocorticoids are clinically used also for peripheral nerve injury, to inhibit inflammations and ameliorate edema. Injection of Dex into the injured peripheral nerve is reported to accelerate neuroregeneration [118]; furthermore, it can delay the development of pain sensitivities and allodynia [119]. Conversely, the activation of glucocorticoid receptors in the spinal cord is reported to enhance the pain behavior of neuropathic pain in the experiment of intrathecal injection of glucocorticoids [120]. Given these complicated effects of glucocorticoids in the CNS and at the site of injury, the administration route of this drug should be carefully considered for application in the case of peripheral nerve injury.

#### 5.1.2. Enhancing the Neuroprotective Function of Microglia/Macrophages

Certain cytokines have been reported to modify the neuroprotective effects of microglia/macrophages. The administration of granulocyte macrophage colony-stimulating factor (GM-CSF) in spinal cord injury has been shown to improve motor function [121] and to help with long-term recovery, accompanied by the upregulation of growth-associated protein 43 (GAP43) by reducing injury-induced neuronal loss [122]. Subcutaneous injection of a mixture of IL-3 and GM-CSF prevents TBI-induced neuronal loss in a rat TBI model and greatly improves motor function after brain injury [90]; moreover, it significantly suppresses neurodegeneration in a model of Parkinson’s disease [123]. In addition, previous reports have shown that IL-3 and GM-CSF can enhance the expression of Bcl-xL, an antiapoptotic factor, in neuronal cells [124,125], which is indicative of their direct effects on neuronal cells. However, as microglia and macrophages also express receptors for these cytokines [90], the cytokines should act on these cells. Indeed, IL-3 and GM-CSF have been shown to induce microglial activation [126] and enhance their survival [127], respectively, by activating the JAK4/STAT5 signaling pathway. The neuroprotective effects of IL-3 and GM-CSF appear to be at least partly attributable to the induction of microglia and macrophage polarization to neuroprotective phenotypes. Cells treated with the cytokines exhibited a significant increase in the expression of neuroprotective factors, such as IGF-1 and HGF, but not of proinflammatory cytokines, such as IL1β [90,123].

IL-3 and GM-CSF have been challenged for the treatment of peripheral nerve injury and neuropathic pain. The administration of IL-3 prevented the loss of motor neurons in the spinal cord after peripheral nerve axotomy [128]. GM-CSF increases the accumulation of macrophages at the site of peripheral nerve injury, thereby enhancing the likelihood of nerve regeneration by increasing the production of neurotrophic factors, such as BDNF [129]. However, because BDNF induces neuropathic pain, GM-CSF and it’s signaling pathways may aggravate arthritic pain [130,131]. Hence, treating neuropathic pain after peripheral nerve injury by inhibiting GM-CSF-mediated signals has also been investigated [132,133]. These results suggest that the therapies for neuropathic pain and nerve regeneration should be developed separately. In addition, not only cytokines but also chemokines have been investigated for their potential as therapeutic agents, and these small proteins warrant further investigation [134].

### 5.2. Controlling Metabolism of Microglia/Macrophages

**Figure 3:**
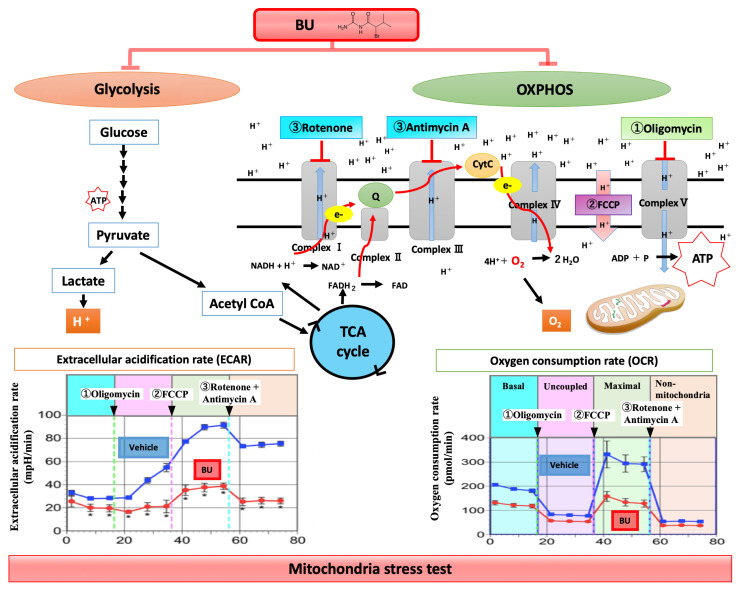
Effects of BU on cell metabolism. The effects of BU on cell metabolism were investigated in primary microglial cells and macrophages using the Seahorse Mito-Stress Test, which assesses OCR and ECAR. In the Mito-Stress Test, to evaluate mitochondrial respiration and glycolysis, various mitochondrial function inhibitors (1, Oligomycin; 2, FCCP; 3, Rotenon and antimycin) are automatically and sequentially added to the cells. BU inhibited mitochondrial ATP production (OXPHOS) but did not affect mitochondrial membrane permeability or coupling efficacy (ATP production divided by basal respiration). BU also prevented the compensatory enhancement of glycolytic activity after the inhibition of mitochondrial ATP synthase (Oligomycin). Based on these results, BU seems to inhibit both OXPHOS and glycolytic pathways without causing mitochondrial dysfunction.

One recently envisaged therapeutic strategy is epigenetic modulation by controlling the metabolism of microglia/macrophages. Bromovalerylurea (BU; C_6_H_11_BrN_2_O, CAS: 496-67-3) is a hypnotic/sedative agent that was developed more than a century ago. It is currently rarely used because of its weaker action as a hypnotic/sedative and dependency compared with newer agents, such as benzodiazepines. BU inhibits excessive inflammation and improves viability in a rat sepsis model prepared using a cecal ligation and puncture method [135]. Moreover, it prevents dopaminergic neuron loss in the substantia nigra pars compacta and improves motor functions in a rat model of Parkinson’s disease developed using 6-hydroxydopamine [111]. It reduced tissue loss and improved cognitive function in a rat stab-wound brain injury model [70]. The anti-inflammatory effect of BU on LPS-treated primary microglia is as strong as that of Dex [136]. BU does not inhibit the nuclear translocation of NFκB but suppresses the phosphorylation of JAK1–STAT1 and the expression of IRF1 [135]. In TBI models, BU inhibited the production of mitoROS and proinflammatory mediators such as iNOS, IL1β, TNFα, and IL6 in microglia and macrophages isolated from the injured brain, thus reducing brain inflammation and oxidative damage [70]. To investigate the effects of BU on the metabolism of cultured primary microglia and macrophages, various inhibitors of mitochondrial metabolism (oligomycin, FCCP, and Rotenon and antimycin) were administered in this order (Figure 3), and changes in oxygen consumption rate (OCR; as an index for OXPHOS) and extracellular acidification rate (ECAR; as an index for glycolysis) were measured as a mitochondria-stress test at each time point. As shown in Figure 3, BU inhibited mitochondrial ATP production (OXPHOS), but did not affect mitochondrial membrane permeability or coupling efficacy (ATP production divided by basal respiration); it also prevented the compensatory enhancement of glycolytic activity after the inhibition of mitochondrial ATP synthase. Therefore, BU inhibits both the OXPHOS and glycolytic pathways without causing mitochondrial dysfunction, thus inhibiting the cellular changes to the proinflammatory phenotype by suppressing cellular metabolism (oxygen requirement), as a hypnotic sedative, but without suppressing the neuroprotective effects of microglia and macrophages [70]. As this effect has recently been revealed, the detailed mechanisms such as intracellular signal cascades are still under investigation. A clinical trial will also be necessary to provide further insight.

Another featured therapeutic agent for brain inflammation is mitochondrial division inhibitor 1 (Mdivi-1), a dynamin-related protein 1 (DRP1) inhibitor. Mitochondrial dysfunction is a common feature in the pathophysiology of the injured CNS. Mitochondria ordinarily undergo repeated fission and fusion to maintain metabolic homeostasis and cellular health. Under normal conditions, the balance of fission and fusion is important for the reorganization of mitochondrial components, removal of damaged material, and maintenance of healthy mitochondria [137,138]. The mitochondrial function is disrupted by the loss of balance between fission and fusion. DRP1 activated by TLR4 signaling increases the mitochondrial fission of microglia and induces metabolic reprogramming from OXPHOS to glycolysis, which subsequently leads to proinflammatory activation [139,140]. Mdivi-1 normalizes OCAR and ECAR by suppressing the mitochondrial fission and suppressing the production of mitoROS and proinflammatory mediators from microglia stimulated by TLR4. Mdivi-1 reduces TBI-induced cell death and morphological change of mitochondria and ameliorates behavioral deficits and brain edema [141]. However, Mdivi-1 has been reported to have no effects on the expression of neuroprotective factors in a brain inflammation model produced by intraperitoneal injections of IL1β [140].

The homeostatic condition of microglia themselves may need to be maintained for them to play their roles in the maintenance of homeostasis. BU and Mivid-1 are believed to normalize excessive proinflammatory reactions by returning metabolism that is shifted toward glycolysis to its original state. Therefore, these agents may be applicable at the hyperacute phase but not acute to the subacute phase when the proinflammatory reaction gradually settles.

In this section, we have focused on the therapeutic strategies for preventing the expansion of damaged areas due to secondary inflammation in TBI and infarction. In the future, regenerative medicine may be the most promising therapy for brain injury or infarction. For instance, induced pluripotent stem (iPS) cells have been used for brain infarction and have shown definite effects in several experiments using rodents [142,143,144,145]. However, such therapies have not yet been applied in clinical settings because they have numerous issues, such as safety, time to transplant, difficulty in normal neural replacement of neural stem cells in various brain cells, including neuronal cells and glial cells, and difficulty in reconstructing normal neural circuits. Therefore, currently, a combination of the abovementioned therapies may be more realistic. Among them, attempts to use BU as a metabolic modulator and Mdivi-1 are novel approaches for treating TBI and infarction. The combination of controlling metabolism at the hyperacute phase and enhancing neuroprotective effects at the acute to subacute phase may become a viable strategy. The discovery of novel biomarkers to evaluate the intracerebral inflammatory states from easily available samples, such as blood, may also be useful.

## 6. Conclusions

Microglia are deeply involved in the maintenance of homeostasis and play critical roles in both the normal and pathological CNS and PNS. The treatments for CNS injury and intractable neuropathic pain targeting microglia and macrophages remain challenging because of the incomplete understanding of both the intra- and extracellular mechanisms that regulate the balance between pro- and anti-inflammatory reactions, as well as of the neuroprotective and neurodestructive actions of the cells. As the extracellular regulatory mechanisms for microglia and macrophages in the pathological CNS, their interactions with other cells, including neurons and glial cells, should be critical for the development of novel interventions aimed at the best control of the cells. Despite the many known signal transduction pathways, the knowledge on the balance among the various pathways that control the nature of microglia remains incomplete. Furthermore, the heterogeneity of the actions of microglia/macrophages remains to be elucidated. The knowledge of their actions in the individual pathological changes has not been integrated to reach a total understanding of microglia and macrophages. This insufficient knowledge may prevent the development of novel interventions aimed at regulating the actions of microglia in the pathological CNS and PNS. Although we did not discuss the results obtained through single-cell RNAseq, precise and detailed knowledge at the single-cell level would provide a breakthrough in this field of research.
